# Gastric outlet obstruction caused by vanishing pylorus following gastric peroral endoscopic myotomy

**DOI:** 10.1055/a-2686-8015

**Published:** 2025-09-04

**Authors:** Abdulrahman Qatomah, Daryl Ramai, Christopher C. Thompson

**Affiliations:** 1 Gastroenterology, Hepatology and Endoscopy, Brigham and Women’s Hospital, Boston, United States; 2 Division of Gastroenterology and Hepatology, King Faisal Specialist Hospital and Research Center, Jeddah, Saudi Arabia

Gastric peroral endoscopic pyloromyotomy (G-POEM) is used to manage gastroparesis that is refractory to conventional therapies 1 2 . Long-term data suggest high overall clinical success (77.5%), with higher success for diabetic gastroparesis (86.5%) 3 . Additionally, the safety of G-POEM has been established, with a very small risk of serious adverse events 4 .

A 68-year-old woman with idiopathic gastroparesis, for which medical and endoscopic therapy had failed, underwent G-POEM ( Fig. 1 ). She was discharged 1 day after the procedure with a liquid diet and twice-daily proton pump inhibitor (PPI). She presented to the emergency department 2 days later with nausea, vomiting, and abdominal pain. Initial vital signs and laboratory blood testing were normal. Subsequent computed tomography scan showed a distended, fluid-filled stomach with radiological evidence of gastric outlet obstruction (GOO) ( Fig. 2 ). A nasogastric tube was placed for gastric decompression.

**Fig. 1:**
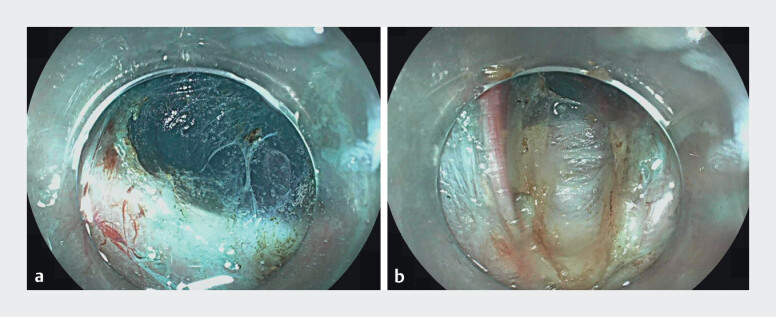
Endoscopic images. **a** Before gastric peroral endoscopic pyloromyotomy (G-POEM). **b** After G-POEM.

**Fig. 2:**
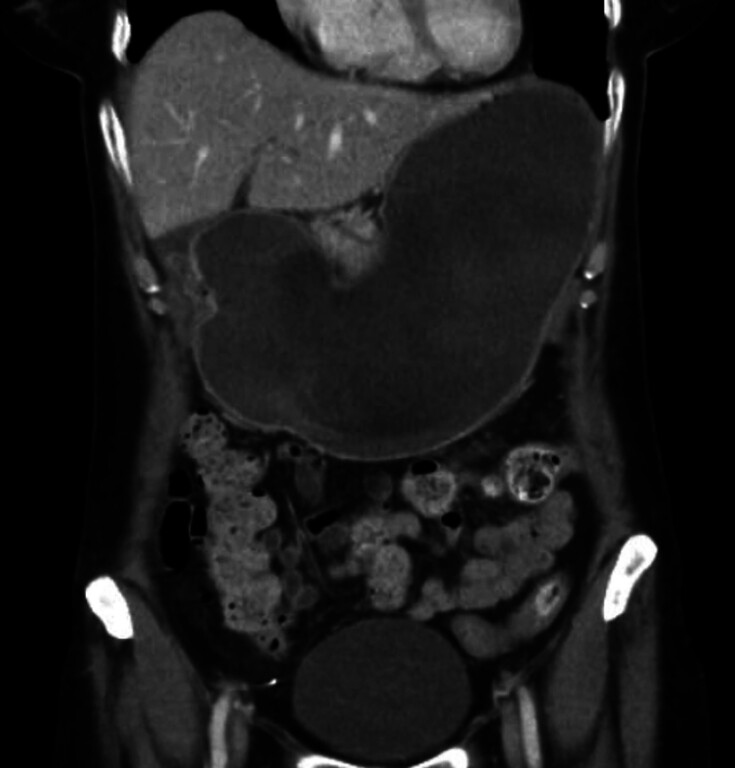
Computed tomography image (coronal) showing the distended, fluid-filled stomach.

Repeat endoscopy was performed, the open mucosotomy was identified, and the tunnel was accessed. There were signs of epithelialization of the submucosa, suggestive of healing. On initial inspection, the pylorus was not visible; however, with careful examination, a pinpoint opening was found and deemed to be the pyloric rim ( Fig. 3 **a** ). A guidewire was advanced into duodenum under fluoroscopic guidance. A 15 × 15 mm lumen-apposing metal stent (LAMS) was placed over the guidewire ( Fig. 3 **b** ). The tunnel was re-accessed, and a hemostatic agent was applied ( Fig. 4 ). The patient was able to tolerate liquid and was discharged the following day on PPI therapy and dietary instruction ( Video 1 ).

**Fig. 3:**
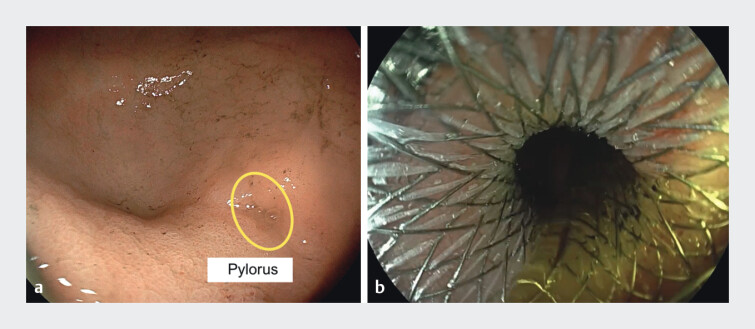
Endoscopic images. **a** Severely stenosed pylorus after gastric peroral endoscopic pyloromyotomy. **b** A lumen-apposing metal stent was placed.

**Fig. 4:**
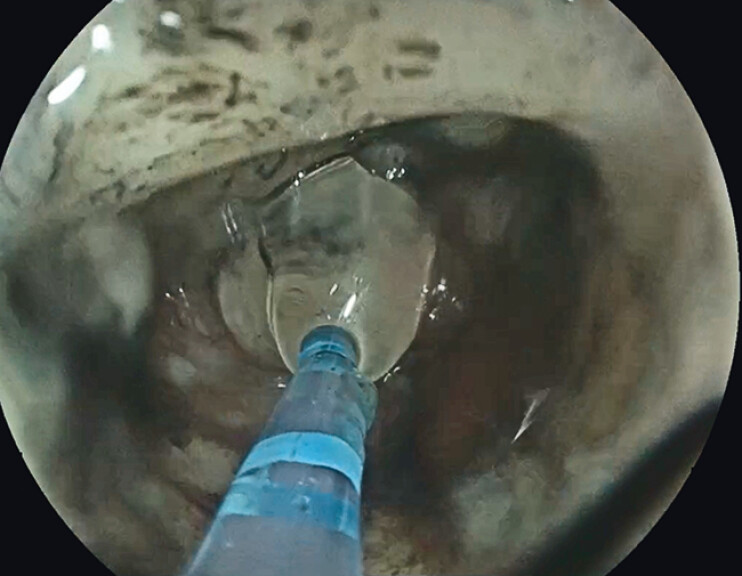
Placement of hemostatic agent within the submucosal tunnel.

Gastric outlet obstruction caused by vanishing pylorus following gastric peroral endoscopic myotomy.Video 1

GOO following G-POEM is rare and has not been described in the literature. Post-procedural inflammation with pyloric edema is a possible etiology in the current case. Careful examination to rule out other etiologies such as accidental suturing of the pylorus during mucosotomy closure is crucial. LAMS placement allows for immediate relief of obstruction and can be subsequently removed after inflammation subsides.

Endoscopy_UCTN_Code_CPL_1AH_2AJ

## Notes

Endoscopy E-Videos https://eref.thieme.de/e-videos
*E-Videos* is an open access online section of the journal *Endoscopy* , reporting on interesting cases and new techniques in gastroenterological endoscopy. All papers include a high-quality video and are published with a Creative Commons CC-BY license. Endoscopy E-Videos qualify for HINARI discounts and waivers and eligibility is automatically checked during the submission process. We grant 100% waivers to articles whose corresponding authors are based in Group A countries and 50% waivers to those who are based in Group B countries as classified by Research4Life (see: https://www.research4life.org/access/eligibility/ ). This section has its own submission website at https://mc.manuscriptcentral.com/e-videos .
